# Practices and Perspectives on Prophylactic Pancreatic Stent Removal Practice: The PIRATE Survey

**DOI:** 10.1055/a-2879-7331

**Published:** 2026-07-16

**Authors:** Marius Zimmerli, Lisette Krassenburg, Enrique de-Madaria, Egle Dieninyte, Paula Ribeiro Sousa, Sophie Schlosser-Hupf, Andreas Wannhoff, Abdullah Murat Buyruk, Lucía Guilabert, Lumir Kunovsky, Mohamed Alboraie, Anna Feiden, Lukas Welsch, Stefanie Reichermeier, Stefan Kahl, Clara Benedetta Conti, Benjamin Misselwitz, Elisabetta Goni, Henriette Heinrich

**Affiliations:** 1 Department of Gastroenterology and HepatologyUniversity Digestive Healthcare CenterBaselSwitzerland; 2 Department of GastroenterologyDr. Balmis General University Hospital-ISABIALAlicanteSpain; 3 Vilnius University, Faculty of MedicineInstitute of Clinical MedicineVilniusLithuania; 4 Vilnius University Hospital Santaros KlinikosVilniusLithuania; 5 Department of GastroenterologyCentro Hospitalar Tondela-ViseuViseuPortugal; 6 Department of Internal Medicine I, Gastroenterology, Hepatology, Endocrinology, Rheumatology and Infectious diseasesUniversity Hospital RegensburgRegensburgGermany; 7 Department of Internal Medicine, Gastroenterology, Haemato Oncology, Diabetology, and InfectiologyRKH Hospital Ludwigsburg, Ludwigsburg HospitalLudwigsburgGermany; 8 Department of GastroenterologyEge University Medical Faculty HospitalİzmirTurkey; 9 2nd Department of Internal Medicine – Gastroenterology and Geriatrics, University Hospital Olomouc, Faculty of Medicine and DentistryPalacky University OlomoucOlomoucCzech Republic; 10 Department of Gastroenterology and Digestive EndoscopyMasaryk Memorial Cancer InstituteBrnoCzech Republic; 11 Department of Surgery, University Hospital Brno, Faculty of MedicineMasaryk UniversityBrnoCzech Republic; 12 Department of Internal MedicineAl-Azhar UniversityNasr CityCairo GovernorateEgypt; 13 Department of Gastroenterology, Diabetology and InfectiologyKlinikum Hanau gGmbHHanauHessenGermany; 14 Department of Gastroenterology and HepatologyKlinikum St Marien AmbergAmbergBYGermany; 15 Hepatology and Infectious DiseasesOtto von Guericke University MagdeburgMagdeburgGermany; 16 Interventional EndoscopyIRCCS San Gerardo dei Tintori Foundation HospitalMonzaLombardyItaly; 17 Department of Medicine IILMU University HospitalMunichGermany

**Keywords:** quality and logistical aspects, training, pancreatobiliary (ERCP/PTCD), ERC topics

## Abstract

**Background and aim** Prophylactic pancreatic stent placement (PPSP) is recommended in high-risk patients undergoing endoscopic retrograde cholangiopancreatography (ERCP) to prevent post-ERCP pancreatitis (PEP). Only a few societies, including the European Society of Gastrointestinal Endoscopy (ESGE), provide guidance on stent management, recommending assessment of spontaneous passage within 5–10 days and endoscopic removal, if retained. However, evidence on real-life adherence to these recommendations remains limited. The primary aim was to assess clinical practices related to PPS removal, methods, timing, and adherence to current guidelines.

**Methods** An online 13-item survey was distributed via professional networks and social media between November 2024 and February 2025 to assess current PPSP and removal practices.

**Results** In all, 322 valid responses were collected (Europe = 285; non-EU = 32; not disclosed = 5). Centers were stratified by annual ERCP volume (procedures/years): <300 (28.3%), 300–500 (31.7%), and >500 (40.1%). Approximately 40% of respondents did routinely place a PPS in high-risk situations. PPSP was most often inserted after >2 pancreatic duct cannulations (83.5%) or papillectomy (58.7%). Straight 5 Fr, 5 cm stents predominated (54% and 85.4%, respectively). Removal practices varied: 62.7% performed endoscopic retrieval, 47% within one week and 41.6% within four weeks; 55% had no fixed follow-up schedule. Complications of nonremoval were reported by 22.7% of the centers—mainly pancreatitis (63%)—yet 96% considered these rare. Overall, 31.7% followed ESGE or internal guidance, while 23.6% reported following no guidance.

**Conclusions** The PIRATE survey reveals that most centers apply heterogenous follow-up removal practices despite presence of guidelines.

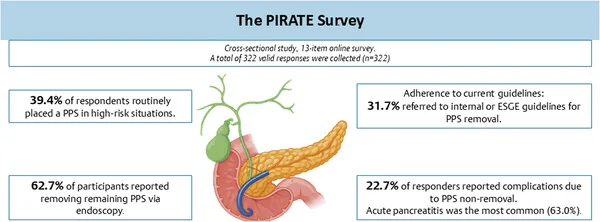

## Introduction

Endoscopic retrograde cholangiopancreatography (ERCP) is an established procedure for the management of biliary and pancreatic disorders worldwide.

Prophylactic pancreatic stent placement (PPSP) is a cornerstone strategy to reduce the incidence and severity of post-ERCP pancreatitis (PEP), occurring in 4–15% of cases. Guidelines recommend PPSP for standard ERCP in patients with recognized patient-related risk factors such as younger age (particularly ≤60 years), female sex, history of acute pancreatitis, and history of prior PEP and procedure-related risk factors such as difficult cannulation (e.g., >3 attempts, prolonged cannulation time, pancreatic duct cannulation more than twice or guidewire passage into the pancreatic duct >1, pancreatic duct contrast injection, pancreatic sphincterotomy, precut (needle-knife) sphincterotomy, endoscopic papillary balloon dilation and failure to clear bile duct stones). 1 2 3 4

Randomized and observational studies consistently demonstrate that PPSP—especially when combined with rectal NSAIDs during ERCP—lowers PEP rates by roughly one-third, yielding a number needed to treat, i.e., 8–10 in high-risk cohorts. 2 3 Observational data indicate that 80–90% of prophylactically placed pancreatic stents (PPSs) dislodge spontaneously within 14 days depending on length and size; however, 10–20% require endoscopic removal. 5 6 7 Complications arising from nonremoval of pancreatic stents are infrequent. Nevertheless, potentially serious adverse events associated with retained stents—including proximal migration, perforation, bleeding, stent fracture, migration, and ductal occlusion—have been reported. 8 Endoscopic retrieval itself carries a ∼3% risk of pancreatitis—particularly when performed early (before 12–24 h) or with larger, internally flanged stents. 9 Despite perceived consensus on PPSP, guidance on the subsequent management of prophylactic stents with regard to removal remains sparse. The European Society of Gastrointestinal Endoscopy (ESGE) and German Society of Gastroenterology (DGVS) specifically recommend that stent passage be assessed radiographically within 5–10 days, with endoscopic retrieval for any retained device, and stipulates a minimum dwell time of 12–24 h to maintain ductal patency, while other guidelines such as the American Society for Gastrointestinal Endoscopy (ASGE) do not give any guidance on PPS removal time. 1 2 3 4

The primary aim of this study was to assess clinical practices related to PPS removal and adherence to current guidelines with regard to PPSP.

## Methods

For this cross-sectional study, a 13-item online REDCap (Vanderbilt University, USA) survey was distributed between November 2024 and February 2025 via social media (LinkedIn, Twitter, Facebook, my UEG community), the UEG Young Talent Group, and selected national gastroenterological societies’ email newsletters **(Supplement 1)** . The introductory text outlined the objective of the survey—to investigate PPSP in nonpancreatic ERC- and included a link to the REDCap survey. Access to REDCap was provided by the Spanish Association of Gastroenterology (AEG) through its AEG REDCap. Four reminders were posted online and via email. No monetary incentives were offered for the study. The 13 multiple-choice questions, partly multianswer questions assessed practice for PPSP placement, removal practice, and timings as well as complications and adherence to guidelines. A categorization into low (300), mid (300–500), and high-volume center (>500) ERCPs per year was chosen to reflect real-world practice patterns, where centers performing more than 300 ERCPs annually are more likely to involve multiple endoscopists and trainees, and therefore have a higher probability of PPSP and removal *.* 10 11

The survey responses were collected and analyzed as deidentified data using IBM SPSS Statistics, version 30.0 (IBM Corp., Armonk, NY, USA). Categorical data were presented as counts and percentages. Group comparisons among the ERCP-volume strata used the chi-square test and Fisher’s exact test. Univariable logistic regression was utilized to interrogate associations. Significance was set at *p* < 0.05.

## Results

### Study Participants

**Fig. 1:**
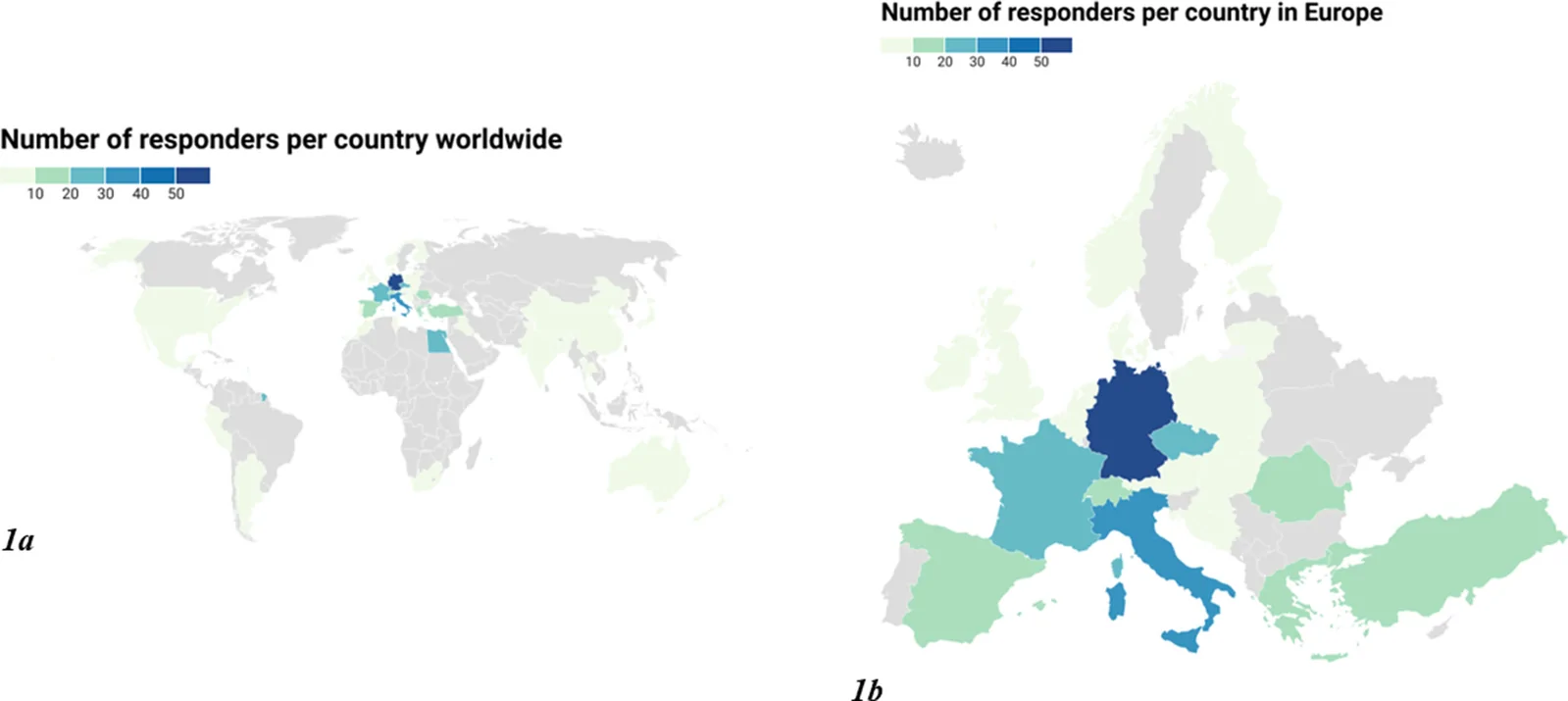
( **a** , **b** ) The maps show the geographical distribution of respondents worldwide ( **a** ) and in Europe ( **b** ).

**Fig. 2:**
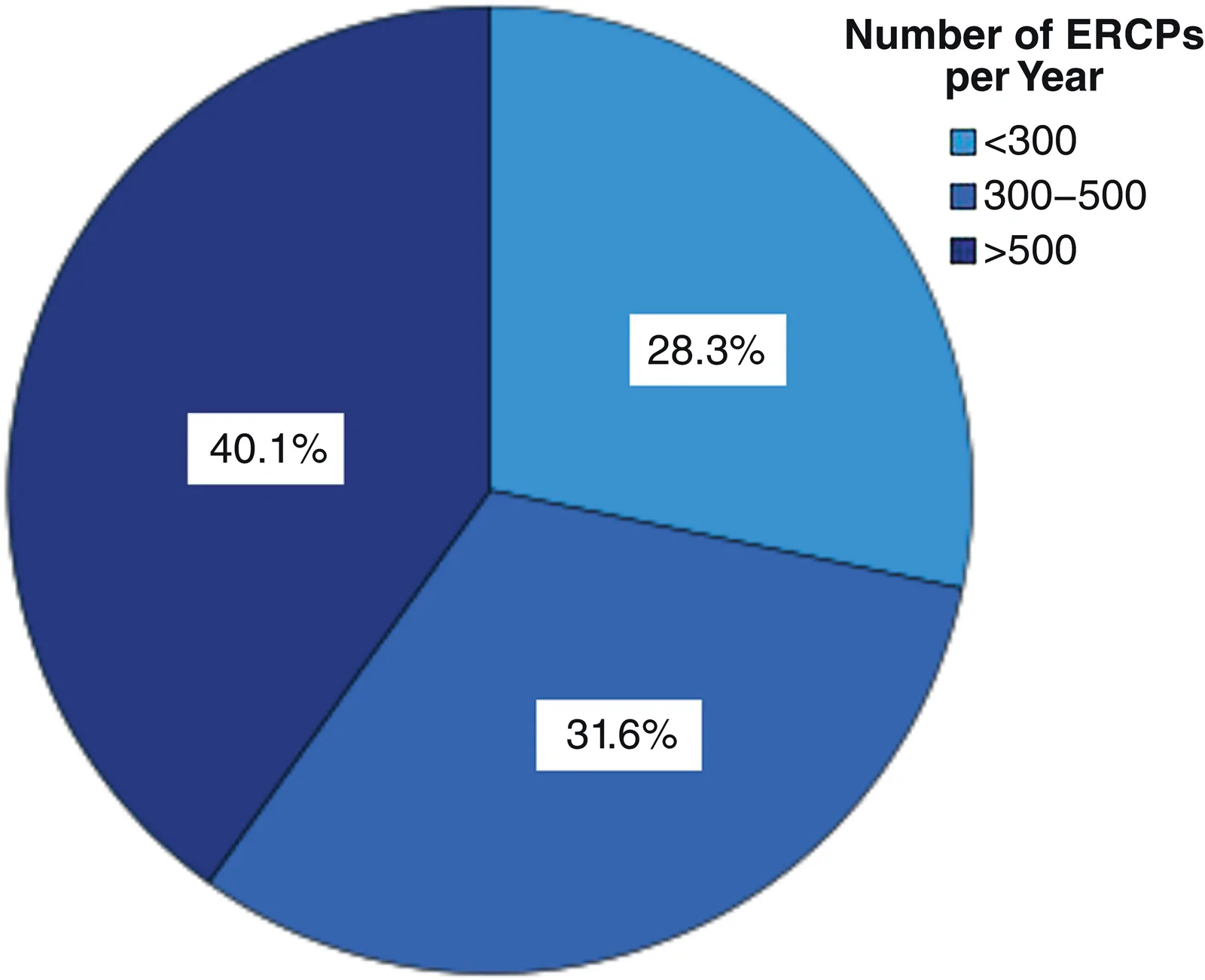
The pie chart illustrates the percentage distribution of ERCP procedures performed annually by the respondents. Each segment represents a specific volume category (e.g., <300 300–500, >500 ERCPs/year), providing an overview of procedural workload among survey participants.

In all, 331 responses were received and 9 were invalid due to incomplete data. In total, 322 responses were received; of those, *n* = 5 did not give their country of origin but provided otherwise complete responses. Totally, 285 (88.5%) of the respondents were based in Europe, while 32 (9.9%) were based in the United States, Oceania, and Asia ( Fig. 1 and b ). About 91 (28.3%) of the respondents reported that their center performed less than 300 ERCPs per year and were therefore classified as low volume centers. About 102 (31.7%) of the centers performed 300–500 ERCPs per year and 129 (40.1%) performed more than 500 ERCPs per year and were therefore classified as high-volume centers ( Fig. 2 ).

### PPSP Practice

When asked if PPSs were placed in all high-risk patients for PEP defined as female gender, pancreatic guidewire passages >2 times, biliary sphincter dilatation, or opacification of the pancreatic duct, only 39.4% of centers reported to generally place a PPS. Overall, 39.4% of centers did place a PPS in high-risk patients.

Respondents from mid-volume centers (OR 0.533; *p* = 0.026 95%; CI 0.31–0.93)) were less likely to place PPS when compared to high- and low-volume centers (OR 0.473, *p* = 0.014 95%; CI 0.26–0.86) ( Fig. 5 ).

When presented with more specific scenarios, 269 respondents (83.5%) reported placing a PPS after more than two unintended pancreatic duct cannulations, and 189 (58.7%) did so following a papillectomy. Accidental opacification and pancreatic endotherapy of the pancreatic duct were seen as a reason for PPSP by 59 (18.3%) of respondents.

Only 56 (17.4%) of centers placed a PPS on initial unwanted cannulation of the pancreatic duct. Overall, 6 (1.9%) never placed a PPS in any of the given scenarios. When comparing central/western European countries (Germany, Italy, France, Spain *n* = 125) with eastern European countries (other European countries, *n* = 155), there was no statistical difference ( *p* = 0.35) with regard to PPS placement and removal practice.

### PPS Specifications

Overall, 173 respondents (53.7%) reported to only use straight stents, while 77 (23.9%) reported to use only mono pigtails. Both stent types were used by 52 (16.1%). There was no difference in the type of stents used when looking at center volume. ( *p* = 0.75)

**Fig. 3:**
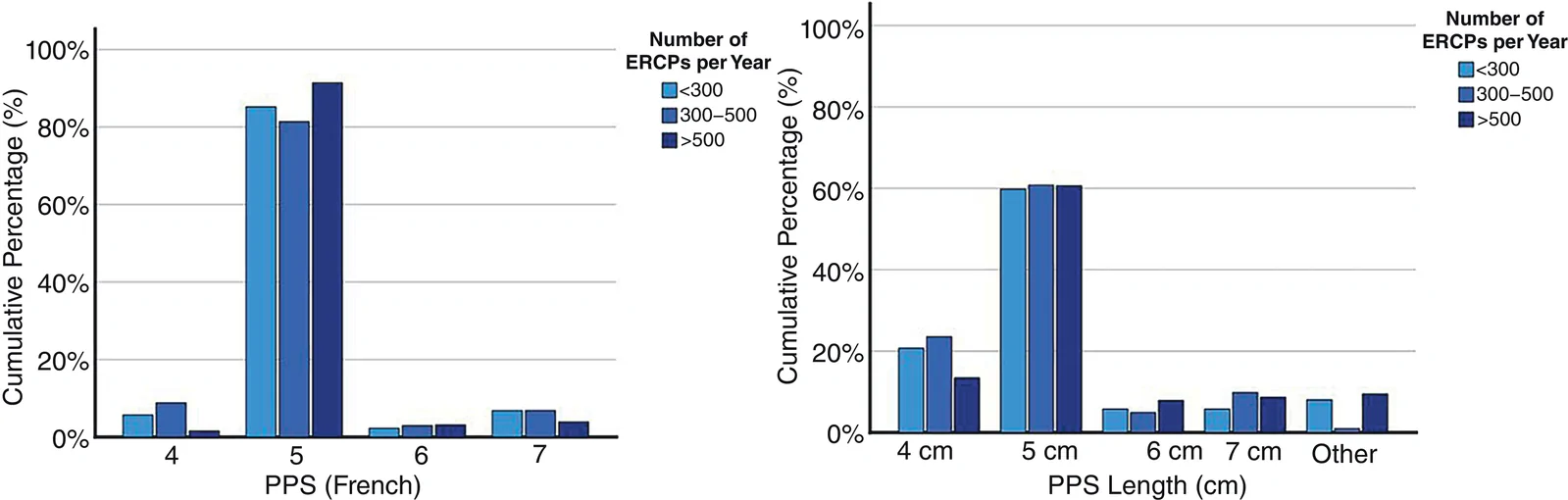
( **a** ) Illustrates the percentage of stent sizes (Fr) by center, expressed as the number of ERCPs per year. ( **b** ) Shows the percentage of stent length (cm) by center, expressed as the number of ERCPs per year.

Other stents, such as biodegradable stents, were used by 4% of the surveyed. Most reported to use 5 French ( *n* = 275 / 85.4%), 5 cm ( *n* = 191; 59.3%) stents. In total, 59 (18.3%) reported to use 4 French stents of varying length ( Fig. 3 a, b ).

### PPS Removal Practice

High volume centers (>500 ERCP per year) reported more often to remove PPS on a regular basis compared to mid- and low-volume centers (71.3% vs. 57.0%; *p* = 0.01, OR 1.88, 95% CI 1.16–3.02). In all, 201 (62.4%) respondents used radiographs to document passage of PPS and only 41(12.7%) used ultrasound. About 202 (62.7%) of participants reported removing remaining PPS via endoscopy. Totally, 132 (41%) of centers gave fixed appointments to remove the stent without a previous check.

Of the respondents removing PPS, the majority, 95 (47.0%), removed the stents within 1 week, while 84 (41.6%) removed stents between one and four weeks. Only a minority of centers – 20 (9.9%) – reported to wait more than 4 weeks until removal. There was no difference in time to stent removal between high-, mid-, or low-volume centers ( *p* = 0.69).

### Complications of PPS Nonremoval

**Fig. 4:**
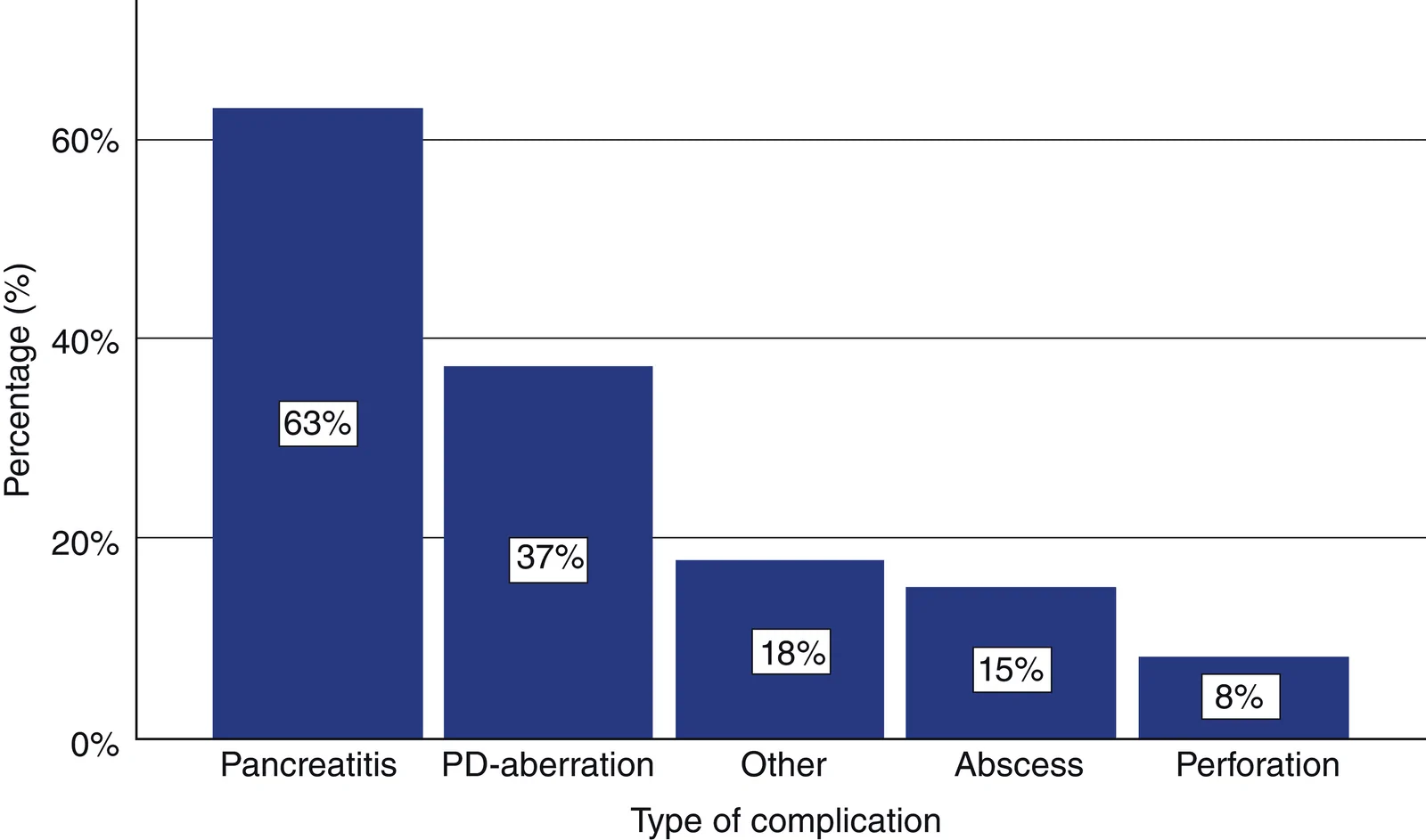
The types of reported complications associated with nonremoval of PPS are described and expressed as percentages (%). Complications of nonremoval of PPS. While acute pancreatitis was the most commonly seen complication, pancreatic duct (PD) aberrations and abscess were also reported. Other complications included stent migration into the bowel or into the pancreatic duct as well as fistula formation.

Totally, 73(22.7%) of centers reported having had complications due to PPS nonremoval. Acute pancreatitis was the most reported complication of nonremoval (63%) ( Fig. 4 ). There was no difference in complications when looking at center volume ( *p* = 0.42).

**Fig. 5:**
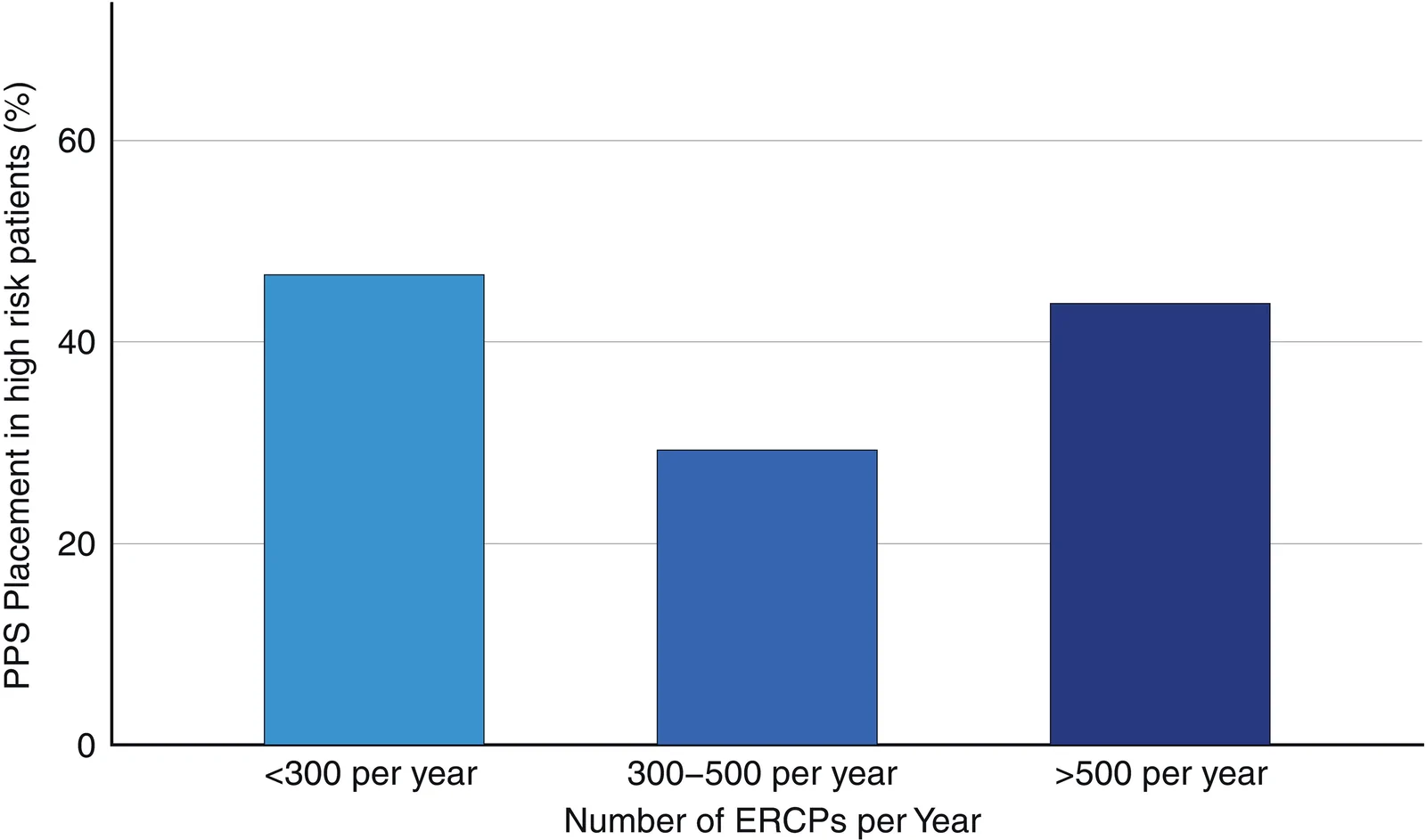
Percentage (%) of PPS placement in high-risk patients according to center volume (number of ERCPs per Year). The bar charts show that low- and high-volume centers have the highest PPSP rates.

The respondents who reported complications ( *n* = 73) perceived these as rare.

### Guidance Followed for PPS Removal

In all, 76 (23.6%) centers reported that there was no clear guidance available on the topic. Overall, 126 (38.5%) relied on experience to decide whether to remove PPS or not, while 102 (31.7%) referred to internal or ESGE guidelines for PPS removal. Totally, 18 (4.7%) referred to other guidance for PPS removal. There was no difference between high- or low-volume centers on which guidance was referred to.

About 244 (75.8%) of respondents believed PPS should be removed, while 23.6% did not deem this necessary. There was no difference between high-, mid-, or low-volume centers with respect to PPS removal.

## Discussion

ERCP remains a cornerstone in the management of pancreaticobiliary diseases. The ESGE guideline on ERCP remains – apart from selected national societies – the only guideline to propose a timeframe for removal and modalities for evaluating spontaneous PPS passage. 1 2 3 4 This survey of 322 respondents offers a large real-world snapshot of PPS practices. Our data suggest a disconnect between evidence-based recommendations and clinical implementation and highlight challenges in follow-up and PPS removal logistics.

Across centers, 5-Fr, 5-cm straight polyethylene stents remained the workhorse device for PPS placement. Although historical studies suggested potential advantages for shorter or smaller-caliber stents in terms of spontaneous passage, a randomized trial of 5-Fr versus 3-Fr pigtail stents demonstrated comparable passage rates (=70%) but significantly easier and faster deployment with 5-Fr stents 5 6 7 12 The ease-of-placement advantage noted in the US survey by Ashat et al. explains the enduring dominance of 5-Fr devices in our data. 13

A key finding of this study is the considerable heterogeneity in PPS follow-up and removal strategies. While 63% of centers reported routine endoscopic removal of retained stents, a substantial proportion relied on radiographic assessment alone, often without direct endoscopist review. This is clinically relevant, as prior data suggest that retained stents may be missed in up to 5% of radiology reports, potentially exposing patients to avoidable complications such as pancreatitis or ductal injury. Furthermore, the widespread reliance on X-ray-based strategies raises concerns regarding cumulative radiation exposure and resource utilization. 8 14 In this context, transabdominal ultrasound (TAUS) represents a promising radiation-free alternative, with emerging evidence supporting its diagnostic accuracy and potential to reduce imaging burden in selected settings. 15 16 17

Current ESGE guidance recommends assessing stent passage at 5–10 days and mandates removal of any retained device after a minimum dwell time of 12 h independent of stent design. The guidelines of the German Society of Gastroenterology (DGVS) only mandate a minimum dwell time of 12–24 h and recommend removal after 3-5 days but without stating a preferred removal modality. 2 4 Although most centers in our cohort adhered to this timeframe, deviations were observed, including delayed removal beyond four weeks and the absence of systematic follow-up in a subset of centers.

Data on the optimal timing of removal and actual spontaneous passage rates are sparse: while immediate removal is discouraged due to the increased risk of pancreatitis. 9 The data are unclear with regard to the optimal timeframe as well as the overall necessity to remove PPS. 5 17 18 19 20 Chahal et al. showed a high spontaneous passage rate of 98% over two weeks with 5 Fr unflanged short PPS. Another study demonstrated similar pass rates for 5-French stents with no increase in complications when retained for more than 10 days. 5 7 These findings likely reflect both the limited granularity of existing guidelines and reliance on individual clinical experience. 21

About 22.7% of centers reported at least one complication of nonremoval – predominantly pancreatitis (67%), perforation (12%), and ductal aberrations (39%), which was independent of center size. The MAUDE analysis corroborates these findings, listing inflammation, embedded stents, and occlusion as the most common patient-level adverse events. 8 22

PEP remains the most common ERCP adverse event, with incidence rates ranging from 4% to 10%. 2 A range of prophylactic measures have been adopted into practice, most notably the use of PPS, rectal nonsteroidal anti-inflammatory drugs (NSAIDs), and periprocedural intravenous (i.e.) hydration. ESGE guidelines advocate the universal administration of rectal NSAIDs for all ERCP patients without contraindications, supplemented by PPSP and aggressive iv hydration with Ringer’s lactate in high-risk individuals. 2 3 4

Importantly only 39.4% of respondents reported routinely placing a PPS in high-risk patients for PEP despite high-quality evidence showing that PPSP and indomethacin prevent PEP in high-risk patients. 10 23 The low rate of PPSP in high-risk patients seems striking but is reflected in recent literature where stenting rates range from 6–50% to 30–50% even in high-risk scenarios. Several factors may explain this observation. First, increasing reliance on pharmacologic prophylaxis – especially rectal indomethacin – may lead some operators to deprioritize stent placement despite evidence suggesting superior efficacy of combined strategies. Second, technical challenges and the non-negligible failure rate of PPS placement may discourage its routine use. Third, concerns regarding cost, procedure time, and the need for follow-up interventions may further limit uptake. Finally, variability in risk perception and patient selection likely contributes to inconsistent implementation of guideline-recommended prophylaxis. 10 23 24

Interestingly, both high- and low-volume centers reported higher rates of PPSP than mid-volume institutions. This suggests that PPS use is not purely a function of procedural experience or annual ERCP numbers. In smaller centers, greater caution and adherence to prophylactic measures may reflect risk-avoidance strategies.

These results partially confirm findings from the US-based national survey by Ashat et al., where high annual ERCP volume was independently and on multivariate analysis strongly associated with increased PPS use. 13

Lower utilization of PPS among mid-volume centers observed in the PIRATE study may reflect several barriers: lack of confidence and expertise in PPSP, variations in the complexity of patients referred to these centers, limited availability of stents, and concerns regarding cost-effectiveness. Furthermore, PPSP fails in 4–10% of cases and failed placement itself increases the risk of pancreatitis and could therefore prohibit stent placement of PPS in risk situations. 1 25 While we did not interrogate indomethacin use in our survey, the work by Asat et al. reported a tendency in lower volume centers to rely exclusively on indomethacin, which may inadvertently deprioritize PPS. 1 13 25

Our survey methodology introduces several caveats. As the focus of our questionnaire was removal of PPS, question design on placement practice could be leading to ambiguous replies. Nevertheless, reported stenting rates are well within reported PPSP rates. 10 24 Convenience sampling via professional networks may over-represent academically engaged centers and under-sample low-resource settings. Response bias and recall bias are inherent to self-reported practice surveys. Although we stratified by ERCP volume, multivariable adjustment for endoscopist expertise, case-mix, and institutional resources was not possible. The cut-off for low-, mid- and high-volume centers was chosen to reflect real-world practice with multiple endoscopists and trainees. Specifically, the >300 ERCP/year threshold has been associated with a transition toward centers involving multiple endoscopists, which may influence both PPS placement and follow-up practices. The additional distinction of very high-volume centers (>500 ERCP/year) was introduced to better capture centers with extensive procedural experience and expertise, which may not be adequately differentiated in binary classifications used in prior studies. This stratification allows for a nuanced interpretation of practice patterns and may explain the nonlinear relationship observed between center volume and PPS utilization in our cohort.

Finally, we could not link practices to concomitant indomethacin use, patient-level outcomes, or the use of flanged or unflanged PPS due to questionnaire design.

## Conclusion

The PIRATE survey highlights considerable heterogeneity in PPS follow-up and removal and shows lack of evidence to inform rational clinical practice with regard to removal policy. Only few guidelines specify dwell time and removal practice regarding specific stent types due to lack of data, which could explain the heterogenous practice for removal and follow-up of PPS.

Prospective observational studies should further investigate the effect of stent design – flanged vs. unflanged, length, and size – on spontaneous pass rates and occurrence of complications. These findings provide clinically relevant evidence to refine guideline recommendations on the necessity of PPS removal. A more individualized strategy may reduce unnecessary radiologic imaging and repeat endoscopic procedures, optimize healthcare resource utilization, and ultimately improve patient comfort, safety, and quality of care.

## Notes

Note This article was changed according to the Correction on 18.09.2026.
**Correction**
In the above-mentioned article the name of the author Clara Benedetta Conti was corrected. This was corrected in the online version on 18.09.2026.

## Supplementary Material

Ergänzendes Material Supplementary Material
