# Correction: Practices and Perspectives on Prophylactic Pancreatic Stent Removal Practice: The PIRATE Survey

**DOI:** 10.1055/a-2956-4815

**Published:** 2026-09-30

**Authors:** Marius Zimmerli, Lisette Krassenburg, Enrique de-Madaria, Egle Dieninyte, Paula Ribeiro Sousa, Sophie Schlosser-Hupf, Andreas Wannhoff, Abdullah Murat Buyruk, Lucía Guilabert, Lumir Kunovsky, Mohamed Alboraie, Anna Feiden, Lukas Welsch, Stefanie Reichermeier, Stefan Kahl, Clara Benedetta Conti, Benjamin Misselwitz, Elisabetta Goni, Henriette Heinrich

**Affiliations:** 1 Department of Gastroenterology and Hepatology 572292 University Digestive Healthcare CenterBaselSwitzerland; 2 Department of Gastroenterology 16802 Dr. Balmis General University Hospital-ISABIALAlicanteValencianaSpain; 3 Institute of Clinical MedicineFaculty of MedicineVilnius UniversityVilniusVilnius CountyLithuania; 4 68626 Vilnius University Hospital Santaros KlinikosVilniusVilnius CountyLithuania; 5 Department of GastroenterologyCentro Hospitalar Tondela-ViseuViseuPortugal; 6 Department of Internal Medicine IGastroenterology, Hepatology, Endocrinology, Rheumatology and Infectious diseasesUniversity Hospital RegensburgRegensburgGermany; 7 Department of Internal MedicineGastroenterology, Haemato Oncology, Diabetology, and Infectiology 363255 RKH Hospital Ludwigsburg, Ludwigsburg HospitalLudwigsburgGermany; 8 Department of Gastroenterology 323336 Ege University Medical Faculty HospitalIzmirİzmirTurkey; 9 2nd Department of Internal Medicine– Gastroenterology and GeriatricsUniversity Hospital Olomouc, Faculty of Medicine and Dentistry 48207 Palacky University OlomoucOlomoucOlomouc RegionCzech Republic; 10 Department of Gastroenterology and Digestive Endoscopy 37748 Masaryk Memorial Cancer InstituteBrnoSouth Moravian RegionCzech Republic; 11 Department of SurgeryUniversity Hospital Brno, Faculty of Medicine 37748 Masaryk UniversityBrnoSouth Moravian RegionCzech Republic; 12 Department of Internal Medicine 68820 Al-Azhar UniversityNasr CityCairo GovernorateEgypt; 13 Department of Gastroenterology, Diabetology and Infectiology 39790 Klinikum Hanau gGmbHHanauHessenGermany; 14 Department of Gastroenterology, Diabetology and Infectiology 9146 Klinikum St Marien AmbergAmbergBYGermany; 15 Hepatology and Infectious Diseases 9376 Otto von Guericke University MagdeburgMagdeburgSachsen-AnhaltGermany; 16 Interventional Endoscopy 9265 IRCCS San Gerardo dei Tintori Foundation HospitalMonzaLombardyItaly; 17 Department of Medicine II 9183 LMU University HospitalMunichBYGermany



In the above-mentioned article the name of the author Clara Benedetta Conti was corrected. This was corrected in the online version on 18.09.2026.

