# Supplementary material for: Practices and Perspectives on Prophylactic Pancreatic Stent Removal Practice: The PIRATE Survey
Source: Endosc Int Open. 2026 Jul 16;14:a28797331. doi: 10.1055/a-2879-7331 (PMC13494744; doi:10.1055/a-2879-7331)
Supplement: Supplementary file 1 [file 10-1055-a-2879-7331_29585200.docx]

**Supplement 1:**

**PIRATE survey Questionnaire:**

1. In which country do you work?

Open answer

Drop down European countries, USA, Japan, India	________________________

2. How many ERCs do you carry out in your institution per year?
A. <300 per year
B. 300-500 per year
C. >500 per year
3. In which situations are prophylactic pancreatic stents placed in your institution? (multiple answers possible)
A. In all high risk patients* (defined as Previous PEP, female gender, pancreatic guidewire passages > 2, biliary balloon sphincter dilatation)
B. unwanted PD canulation >2x
C. every time the guidewire passes into the PD
D. Never
E. In selected cased
F. After papillectomy
4. What kind of prophylactic pancreatic stents are used in your institution? (multiple answer possible)
A. Monopigtails
B. Straight stents
C. Other (please explain)
5. Which french of pancreatic stents are usually placed in your institution for prophylactic pancreatic stenting
A. 4
B. 5
C. 6
D. 7
6. Do you regularly remove prophylactic pancreatic stents with gastroscopy?
A. Yes
B. No
7. If yes after how much time?
A. 1 Week or less
B. Between1 week and 4 weeks
C. More than 4 weeks
8. How do you check for prophylactic pancreatic stent retention (multiple answer possible)
A. Radiograph
B. Fixed appointment to remove the stent without previous check
C. Transabdomnal Ultrasound
D. Other (please explain)
9. Have you experienced complications due to non-removal of pancreatic stents in your institution
A. Yes
B. No
10. If yes which complication (more than one option possible)
A. Perforation
B. Pancreatitis
C. Abcess
D. Pancreatic duct aberations
E. Other (Open text) (open text if you select this one)
11. If yes how often did those complications occur
A. Rarely
B. Often
12. To which guidance do you refer for removal or non removal of pancreatic stents
A. Open answer (Free Text)
B. Internal guidance
C. Experience
D. There is no guidance
13. In your opinion, is it necessary to remove the prophylactic pancreatic stents?
A. Yes
B. No
